# Selective plant–microbe communication shapes microbiome-assisted immunity

**DOI:** 10.1093/ismejo/wrag270

**Published:** 2026-10-17

**Authors:** Muhammad Rahil Afzal, Sisi Lin, Mingjie Liang, Linhong Jin, Ming Chang, Libo Zhang

**Affiliations:** State Key Laboratory of Green Pesticide, Guizhou University, Guiyang 550025, China; Key Laboratory of Soybean Disease and Pest Control (Ministry of Agriculture and Rural Affairs), College of Life Sciences, Nanjing Agricultural University, Nanjing 210095, China; State Key Laboratory of Green Pesticide, Guizhou University, Guiyang 550025, China; State Key Laboratory of Green Pesticide, Guizhou University, Guiyang 550025, China; Key Laboratory of Soybean Disease and Pest Control (Ministry of Agriculture and Rural Affairs), College of Life Sciences, Nanjing Agricultural University, Nanjing 210095, China; State Key Laboratory of Green Pesticide, Guizhou University, Guiyang 550025, China

**Keywords:** *Bacillus*, disease resistance, lipopeptide, microbiota, plant–microbe interaction, rhizosphere bacteria

Plants live in close association with highly diverse microbial communities that include both pathogens and beneficial microbes [1, 2]. While canonical plant immunity has largely been studied through the framework of pattern-triggered immunity (PTI) and effector-triggered immunity (ETI) [3, 4], these models primarily explain how plants recognize and restrict invading pathogens. By contrast, how plants selectively engage beneficial microbes to enhance disease resistance remains incompletely understood. This does not imply that beneficial plant–microbe interactions lack mechanistic foundations; rather, the mechanisms by which plants selectively assemble and perceive disease-protective microbiota beyond dedicated symbioses remain less resolved [5].

This presents a fundamental challenge for plant immunity: protective microbes must be recruited and perceived in ways that strengthen host defense, while plant immunity must simultaneously be balanced against the growth costs associated with defense activation [6]. Pathogen infection can trigger changes in root exudation and the recruitment of beneficial or disease-suppressive microbes [7], while interactions with beneficial microbes can establish primed immune states that enhance subsequent defense without constitutive activation of costly defenses [8]. Increasing evidence suggests that plants actively shape these interactions through chemically mediated communication with their associated microbiota [7].

Two recent studies provide additional mechanistic insights into how this can work. Zeng *et al.* demonstrate that pathogen-triggered immune signaling in aerial tissues induces root exudation of heptadecanoic acid, which selectively enriches protective rhizosphere bacteria [9]. Gilliard *et al.* reveal that plants perceive a *Bacillus*-derived lipopeptide through membrane biophysical remodeling rather than canonical receptor-mediated recognition [10]. Although these mechanisms have been demonstrated in rice (*Oryza sativa*) and *Arabidopsis thaliana* respectively, together they provide complementary mechanistic examples that motivate a testable hypothesis about whether pathogen-triggered microbiota recruitment and microbial-signal perception can be functionally connected.

Plants do not passively coexist with rhizosphere microbes but actively shape microbial community assembly through dynamic root exudation. Increasing evidence indicates that pathogen infection can systemically reprogram rhizosphere composition, suggesting that microbiota assembly constitutes an integral component of plant immune responses [11]. However, the molecular mechanisms linking immune perception in aerial tissues to selective recruitment of protective microbes in roots have remained largely unclear.

Zeng *et al.* now provide a mechanistic framework for this process in rice. Upon infection by the foliar pathogen *Magnaporthe oryzae*, immune signaling is transmitted from leaves to roots through the OsMKK4–OsMPK6–OsWRKY24/53/70 cascade, which directly activates expression of the fatty acid biosynthetic gene *3-KETOACYL-COA SYNTHASE 2* (*OsKCS2*) [9]. This pathway promotes the biosynthesis and root secretion of heptadecanoic acid, a specific metabolite that selectively enriches *Bacillus* species within the rhizosphere. The recruited *Bacillus* strains subsequently enhance host resistance through both direct antagonism of pathogen growth and induction of systemic resistance [9].

This study reveals an unexpectedly high degree of chemical specificity in microbiota recruitment [9]. The structural analog palmitic acid does not exhibit equivalent recruitment activities, suggesting that plants can selectively shape protective microbiota through defined metabolite signals rather than through broad changes in root exudation. Such specificity may allow plants to enrich beneficial microbes while minimizing disruption to resident microbial communities.

Root exudate-mediated microbiota assembly can function as a regulated component of systemic immunity rather than as a passive outcome of infection (Fig. 1). Recruitment is linked to pathogen-triggered immune signaling rather than simply to disease progression, implying that plants may establish protective microbial associations at early stages of pathogen challenge.

**Figure 1:**
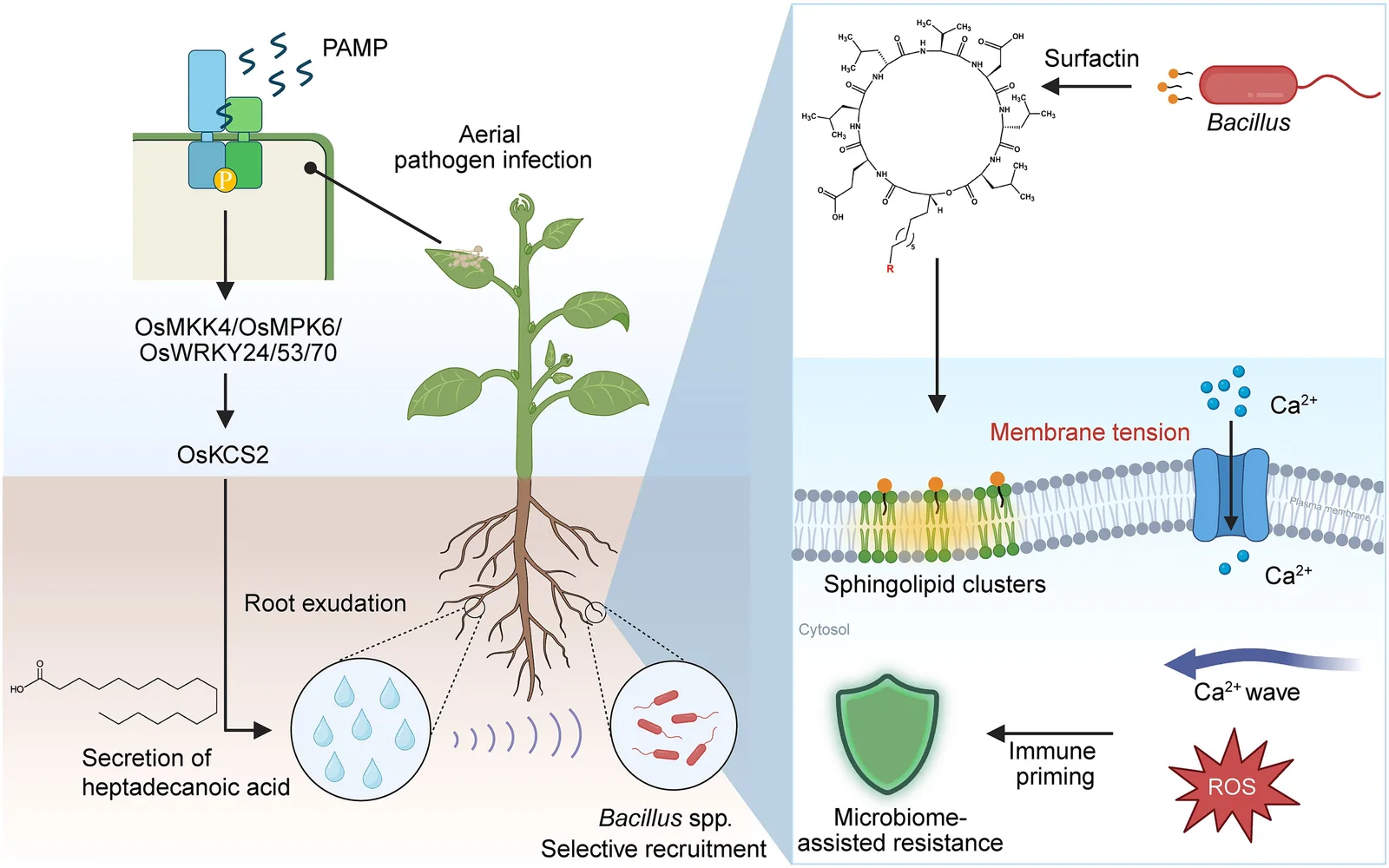
A proposed model for selective recruitment and perception in plant–microbe communication. Upon pathogen infection in aerial tissues, pathogen-associated molecular patterns (PAMPs) are perceived via pattern-recognition receptors (PRRs), and immune signaling is systemically transmitted from leaves to roots through the OsMKK4–OsMPK6–OsWRKY24/53/70 cascade, promoting biosynthesis and secretion of heptadecanoic acid. This metabolite selectively enriches beneficial *Bacillus* species within the rhizosphere, thereby assembling a protective microbiota. Beneficial microbes subsequently produce signaling molecules such as surfactin, which is perceived through plasma membrane biophysical remodeling rather than canonical PRR-mediated recognition. This process activates early immune responses, including calcium (Ca^2+^) signaling, reactive oxygen species (ROS) production, and immune priming, ultimately enhancing disease resistance while minimizing excessive defense activation.

Recruitment of beneficial microbes is only one side of the communication process. To translate the presence of recruited microbes into enhanced immunity, plants must also perceive microbial-derived signals in ways that activate defense without triggering the full costs associated with pathogen-induced immunity. A central, largely unanswered question is therefore how plants distinguish beneficial microbial cues from pathogenic signals while maintaining immune homeostasis.

Recent work by Gilliard *et al.* provides an important conceptual advance by showing that plant perception of beneficial microbes may operate through mechanisms distinct from canonical PTI [10]. The study demonstrated that surfactin, a cyclic lipopeptide produced by *Bacillus* species, activates immune responses in *Arabidopsis* largely independently of canonical pattern-recognition receptor (PRR) signaling pathways. Instead, surfactin preferentially interacts with plasma membrane (PM) sphingolipids, increasing membrane rigidity and lateral tension. These changes activate mechanosensitive ion channels and trigger early immune outputs, including calcium (Ca^2+^) influx, reactive oxygen species (ROS) production, and membrane depolarization. Root application of surfactin further enhances systemic resistance against the necrotrophic pathogen *Botrytis cinerea*, demonstrating that perception of beneficial microbial signals can effectively prime plant immunity [10].

This work expands the current understanding of plant immune perception (Fig. 1). In canonical PTI, microbial molecules are recognized through highly specific ligand–receptor interactions mediated by cell-surface PRRs [3]. In contrast, the surfactin case points to a different mode of perception, in which biophysical changes in the PM contribute directly to immune activation [10]. In this framework, the PM may function not only as a scaffold for immune receptors, but also as a sensory interface whose lipid composition and mechanical properties contribute directly to immune activation.

Beneficial microbes can induce systemic resistance through immune priming, in which defense responses are potentiated upon subsequent pathogen challenge without constitutive activation of the full defense programme [8, 12]. The key unresolved question is how such priming states are established and maintained at the molecular level. The PM biophysical remodeling mechanism described above identifies one route by which a beneficial microbe-derived signal can activate immunity without relying on the canonical PRR pathways tested in this system [10], although it should not be interpreted as a general explanation for how plants distinguish beneficial from pathogenic microbes. This idea is particularly relevant in the rhizosphere, where plants are continuously exposed to dense microbial communities and must balance immune surveillance with microbiome compatibility.

A central challenge for plants is not simply how to defend against pathogens, but how to selectively recruit and appropriately perceive beneficial microbes within highly complex microbial communities. An important implication of these findings is the possibility of a functional link between microbiota recruitment and microbial-signal perception in plant immunity. Upon pathogen attack, plants actively reshape root exudation profiles to enrich protective microbiota from bulk soil [9]. At a complementary stage, beneficial microbes can produce chemical signals that are perceived through noncanonical immune mechanisms capable of priming defense without inducing excessive immune activation [10]. These processes have been demonstrated in rice and *Arabidopsis*, respectively, and their functional integration within a single plant species remains to be established. The proposed recruitment–perception link should therefore be viewed as a testable conceptual hypothesis rather than an experimentally validated pathway. A critical experimental question is therefore whether the microbes recruited by a pathogen-responsive programme can subsequently provide signals that engage a distinct host perception mechanism, and whether disrupting either step compromises microbiome-assisted resistance. If such coordination exists, it could provide plants with a flexible mechanism for balancing growth and immunity under fluctuating environmental conditions.

Despite these advances, several fundamental questions remain unresolved. First, how plants achieve high specificity during microbiota recruitment remains incompletely understood. Recent studies suggest that chemically distinct root exudates can differentially shape rhizosphere microbial community assembly [13], implying the existence of highly regulated metabolic selection systems. Chemically diverse metabolites have now been implicated in rhizosphere microbiome assembly. Heptadecanoic acid has been demonstrated to function as a recruitment signal in rice [9], whereas dopamine has been implicated in microbiome assembly in *Brachypodium* [14], and salicylic acid-associated signaling has been linked to changes in root-associated microbiota in both rice and *Arabidopsis* [15]. Rather than pointing to a single universal recruitment signal, these findings raise the possibility that plants use chemically distinct, context-dependent metabolites to shape different microbiome configurations. An important question is therefore not simply which metabolite is the recruitment signal, but whether individual metabolites act directly on microbial populations, indirectly through host physiology, or in combination with other exudate components to determine community assembly.

Second, the mechanisms underlying selective perception of beneficial microbes remain largely unknown. The surfactin study suggests that plants may sense this *Bacillus*-derived lipopeptide signal through membrane biophysical changes rather than canonical receptor-mediated recognition [10]. However, it remains unclear how plants distinguish beneficial *Bacillus* strains from pathogens that can likewise alter membrane rigidity or tension. Such discrimination may depend on the lipid partners engaged, the magnitude or duration of the biophysical change, or additional microbial cues associated with surfactin. It may also depend on cellular context, because the same membrane perturbation could produce different outputs depending on PM lipid composition, ion-channel abundance, or the concurrent activation of canonical immune pathways. This raises broader questions regarding whether PM lipid composition, mechanosensitive ion channels, and membrane-associated Ca^2+^ signaling constitute a specialized surveillance system for monitoring microbiota-derived signals. Determining how these variables are integrated will be critical for establishing whether membrane-based sensing can function as a selective filter in complex microbial communities. A major technical challenge will be to determine whether such biophysical sensing is broadly used by plants or represents a signal- and context-specific mechanism. Integrating experiments with selected microbial communities, targeted genetic perturbations, spatial metabolite analysis, membrane-state reporters, and time-resolved microbiome profiling could help establish causal links between microbial signals, host perception, microbiome assembly, and disease resistance.

Finally, these discoveries provide a conceptual framework for developing sustainable disease-control strategies based on microbiome engineering. The use of nonpathogenic elicitors to manipulate plant–microbe communication to regulate plant immunity has already been proposed [12]. The potential conceptual advance here is therefore not the use of nonpathogenic elicitors per se, but the possibility of exploiting the relationship between pathogen-responsive recruitment and subsequent microbial-signal perception. The two studies discussed here suggest a more specific hypothesis: pathogen-responsive recruitment and subsequent microbial-signal perception could, in some contexts, represent functionally coupled outputs of plant–microbe communication, allowing recruited microbes to function not only as antagonists but also as sources of immune-priming signals. Testing it would require, for example, linking pathogen-induced recruitment and microbial signal perception within the same host species, and determining whether genetic or chemical disruption of recruitment alters subsequent microbial-signal perception and disease resistance.

This perspective also shifts the engineering objective from simply supplying an elicitor or recruitment metabolite toward manipulating the host–microbiome interaction at multiple causal steps. Metabolites or microbial signals identified in these studies could serve as entry points for such interventions, but their utility will depend on whether their effects can be reproduced in defined and field-relevant microbial communities. Translation to the field will, however, be constrained by the limited stability of synthetic metabolites in soil and by the high spatiotemporal variability of rhizosphere microbiomes. An additional bottleneck is establishing causal, rather than merely correlative, relationships between plant-derived metabolites, specific microbial populations or functions, and disease resistance in complex communities. Such relationships may be difficult to reproduce across soils, cultivars, developmental stages, and environmental conditions. Addressing these constraints will require the integration of synthetic-community experiments, spatially resolved metabolite measurements, microbial genetics, host genetic perturbation, and field-scale microbiome profiling to determine when and where recruitment–perception coupling contributes to disease resistance. Understanding whether and how plants coordinate selective recruitment and selective perception may ultimately enable rational manipulation of plant–microbe communication networks for durable disease resistance and sustainable agriculture.

## Data Availability

Data sharing not applicable to this article as no datasets were generated or analysed during the current study.
